# Response Assessment in Long-Term Glioblastoma Survivors Using a Multiparametric MRI-Based Prediction Model

**DOI:** 10.3390/brainsci15020146

**Published:** 2025-01-31

**Authors:** Laiz Laura de Godoy, Archith Rajan, Amir Banihashemi, Thara Patel, Arati Desai, Stephen Bagley, Steven Brem, Sanjeev Chawla, Suyash Mohan

**Affiliations:** 1 Departments of Radiology, Perelman School of Medicine at the University of Pennsylvania, Philadelphia, PA 19104, USA; laiz.godoy@pennmedicine.upenn.edu (L.L.d.G.); archith.rajan@pennmedicine.upenn.edu (A.R.); suyash.mohan@pennmedicine.upenn.edu (S.M.); 2 Pathology and Laboratory Medicine, Perelman School of Medicine at the University of Pennsylvania, Philadelphia, PA 19104, USA; amir.banihashemi@pennmedicine.upenn.edu; 3 Neurosurgery, Perelman School of Medicine at the University of Pennsylvania, Philadelphia, PA 19104, USA; thara.patel@pennmedicine.upenn.edu (T.P.); steven.brem@pennmedicine.upenn.edu (S.B.); 4 Abramson Cancer Center, Perelman School of Medicine at the University of Pennsylvania, Philadelphia, PA 19104, USA; arati.desai@pennmedicine.upenn.edu (A.D.); sbagley@pennmedicine.upenn.edu (S.B.); 5 Glioblastoma Translational Center of Excellence, Perelman School of Medicine at the University of Pennsylvania, Philadelphia, PA 19104, USA

**Keywords:** glioblastoma, long-term survival, pseudoprogression, true progression, diffusion tensor imaging, dynamic susceptibility contrast-perfusion MRI

## Abstract

**Purpose:** Early treatment response assessments are crucial, and the results are known to better correlate with prognosis and survival outcomes. The present study was conducted to differentiate true progression (TP) from pseudoprogression (PsP) in long-term-surviving glioblastoma patients using our previously established multiparametric MRI-based predictive model, as well as to identify clinical factors impacting survival outcomes in these patients. **Methods:** We report six patients with glioblastoma that had an overall survival longer than 5 years. When tumor specimens were available from second-stage surgery, histopathological analyses were used to classify between TP (>25% characteristics of malignant neoplasms; *n* = 2) and PsP (<25% characteristics of malignant neoplasms; *n* = 2). In the absence of histopathology, modified RANO criteria were assessed to determine the presence of TP (*n* = 1) or PsP (*n* = 1). The predictive probabilities (PPs) of tumor progression were measured from contrast-enhancing regions of neoplasms using a multiparametric MRI-based prediction model. Subsequently, these PP values were used to define each lesion as TP (PP ≥ 50%) or PsP (PP < 50%). Additionally, detailed clinical information was collected. **Results:** Our predictive model correctly identified all patients with TP (*n* = 3) and PsP (*n* = 3) cases, reflecting a significant concordance between histopathology/modified RANO criteria and PP values. The overall survival varied from 5.1 to 12.3 years. Five of the six glioblastoma patients were MGMT promoter methylated. All patients were female, with a median age of 56 years. Moreover, all six patients had a good functional status (KPS ≥ 70), underwent near-total/complete resection, and received alternative therapies. **Conclusions:** Multiparametric MRI can aid in assessing treatment response in long-term-surviving glioblastoma patients.

## 1. Introduction

Glioblastomas typically exhibit substantial intratumoral heterogeneity in molecular expression, epigenetic and genetic markers [1], metabolism, and neuroimaging patterns [2]. Despite the multidisciplinary standard of care during treatment, including maximal safe resection followed by chemoradiation (NCCN Guidelines) [3], the vast majority of patients develop a new contrast-enhancing lesion within the radiation field within six months post-concurrent chemo-radiotherapy (CCRT) [4,5,6]. This new lesion could represent true tumor progression (TP), reflecting viable neoplastic cells, or pseudoprogression (PsP), consisting of predominantly treatment-related changes, characterized by geographic necrosis, reactive gliosis, and vascular hyalinization [7]. PsP simulates progressive disease on neuroimaging in patients undergoing CCRT, induced by temozolamide-mediated increased vascular leakiness and radiation therapy and stabilizes or resolves spontaneously without further treatment [8,9]. Notably, patients with GBMs harboring O^6^-methylguanine–DNA–methyltransferase (MGMT) promoter methylation are more likely to experience PsP and demonstrate improved overall survival (OS) [9,10]. Accurate differentiation between PsP and TP is essential for appropriate therapeutic decision making and prognostication [11,12].

Glioblastomas carry a dismal prognosis; for example, the median overall survival for newly diagnosed glioblastoma remains 15–17 months, with a 5-year survival rate <5% [10,13]. An accurate assessment of treatment response (TP versus PsP) is crucial for optimal clinical management and improving survival outcomes. However, conventional neuroimaging often fails to accurately distinguish between TP and PsP [12,14]. Nevertheless, advanced MR imaging techniques such as diffusion tensor imaging (DTI) and dynamic susceptibility contrast (DSC) perfusion-weighted imaging (PWI) have shown potential utility in differentiating PsP from TP [15,16].

We previously developed a multiparametric MRI-based prediction model by combining the unique strengths of DTI and DSC PWI-derived parameters in evaluating treatment response with an accuracy of 91% in glioblastoma patients treated with surgery and CCRT [16]. Moving forward, the diagnostic performance of this multiparametric MRI-based prediction model was validated in an independent cohort of glioblastoma patients treated with standard of care (SOC) treatment, i.e., surgery followed by CCRT [17]. This approach has also shown high accuracy in assessing treatment response to anti-EGFRvIII chimeric antigen receptor T cell (CAR-T) therapy [18] and the autologous, tumor lysate-loaded dendritic cell vaccine (DCVax-L) in glioblastoma patients [19].

Generally, long-term survivors of glioblastoma are defined as patients who live for two to ten years (or longer) beyond their initial histological diagnosis [20]. In the present study, patients with glioblastoma who lived more than 5 years were considered as long-term survivors. This threshold was chosen due to its clinical significance and the high number of studies using a similar definition [21,22,23].

In this study, we describe six long-term glioblastoma survivors (> 5 years) and applied our established multiparametric MRI-based predictive model to evaluate treatment response. In addition, we aimed to identify any potential associations among clinical prognostic factors and long-term survival outcomes in this rare cohort of patients with glioblastoma.

## 2. Materials and Methods

### 2.1. Patient Population

The institutional review board (protocol # 829645) approved this study, which was compliant with the Health Insurance Portability and Accountability Act. The inclusion criteria for recruitment in the present study were that all patients had (*i*) a diagnosis of glioblastoma confirmed by histopathological analyses; (*ii*) molecularly confirmed isocitrate dehydrogenase (*IDH*) wild-type; (*iii*) were treated with standard-of-care therapy (surgical resection and CCRT); (*iv*) presented a new enhancing lesion in the radiation field on follow-up MRI at any point after completion of CCRT; (*v*) had available anatomical and physiological neuroimaging (DTI and DSC-PWI); and (*vi*) had an overall survival (OS) longer than 5 years. Based upon the inclusion criteria, a cohort of 6 patients was identified.

### 2.2. MRI Data Acquisition

All patients underwent MRI on a 3T Tim Trio whole-body MR scanner (Siemens, Erlangen, Germany) equipped with a 12-channel phased-array head coil. The anatomical imaging protocol included the axial 3D-T1-weighted magnetization-prepared rapid acquisition of gradient echo (T1-MPRAGE) imaging and axial T2-fluid attenuated inversion recovery (T2-FLAIR) imaging using standard parameters. The postcontrast T1-weighted images were acquired with the same parameters as the precontrast acquisition after injecting a standard dose (0.14 mmol/Kg) of gadolinium-based contrast agent using a power injector (Medrad, Idianola, PA, USA).

### 2.3. Diffusion Tensor Imaging

Axial DTI data were acquired using 30 noncollinear/noncoplanar directions with a single-shot spin echo, echo-planar read-out sequence with parallel imaging by using generalized autocalibrating partially parallel acquisition (GRAPPA) and an acceleration factor of 2. The sequence parameters were as follows: repetition time (TR)/echo time (TE) = 5000/86 ms; number of excitations (NEX) = 3; field of view (FOV) = 22 × 22 cm^2^; matrix size = 128 × 128; in-plane resolution = 1.72 × 1.72 mm^2^; slice thickness = 3 mm; b = 0, 1000 s/mm^2^; number of slices = 40; acquisition time 8 min.

### 2.4. Dynamic Susceptibility Contrast-Perfusion Weighted Imaging

For axial DSC-PWI, a bolus of gadobenate dimeglumine (Multi-Hance; Bracco Diagnostics, Princeton, NJ, USA) was injected with a preloading dose of 0.07 mmol/kg, to reduce the effect of contrast agent leakage on cerebral blood volume (CBV) measurements. A T2*-weighted gradient-echo EPI was used during the second 0.07 mmol/kg bolus of contrast agent for the DSC-PWI. The injection rate was 5 mL/s for all patients and was immediately followed by a flush of saline (total of 20 mL at the same rate). The sequence parameters were as follows: TR/TE = 2000/45 ms; FOV = 22 × 22 cm^2^; matrix size = 128 × 128; in-plane resolution = 1.72 × 1.72 mm^2^; slice thickness = 3 mm; bandwidth = 1346 Hz/pixel; flip angle = 90°; EPI factor = 128; echo spacing = 0.83; acquisition time 3 min and 10 s. Forty-five sequential measurements were acquired for each section.

### 2.5. Image Processing

In-house-developed algorithms were applied to raw DTI data to correct for any motion and eddy current-induced artifacts. Subsequently, pixel-wise mean diffusivity (MD), fractional anisotropy (FA), coefficient of linear anisotropy (CL), planar anisotropy (CP), and spherical anisotropy (CS) maps were generated by using the methods described previously [24,25]. The DSC-PWI data were processed using NordicICE software 4.1.0. (NordicNeuroLab, Bergen, Norway). Briefly, a well-established tracer kinetic model for the first-pass data was applied to obtain CBV maps. To reduce the effects of recirculation, the gamma-variate function, which is an approximation of the first-pass response as it would appear in the absence of recirculation, was fitted to the 1/T2* curves. Subsequently, dynamic curves were mathematically corrected to reduce contrast agent leakage effects. After reducing the effects of recirculation and leakage of the contrast agent, CBV was computed with numeric integration of the curve.

The DTI-derived maps, CBV maps, and T2-FLAIR images were resliced and co-registered to contrast-enhanced T1-weighted images. A semiautomatic approach was used to segment the contrast-enhancing regions of each lesion by using a signal intensity-based thresholding method [24,25]. The median values of DTI metrics (MD, FA, CL, CP, and CS) from the contrast-enhancing regions were measured. In addition, the lower 10th percentile MD values were measured from the enhancing region and were reported as MD_min_. The CBV values from the enhancing regions were normalized using corresponding values from contralateral normal white matter to obtain the relative CBV (rCBV). The top 90th percentile rCBV values were also measured from the enhancing region and were reported as the maximum rCBV (rCBV_max_).

### 2.6. Radiographic Response Assessment Using Modified RANO Criteria

In patients in whom repeat surgery or biopsy was not performed, modified RANO criteria [26] were used to determine the final diagnosis of TP or PsP. The tumor size was determined as the sum of the products of diameters (SPDs) on the post-contrast T1 images. As the modified RANO working group has suggested that radiological response at the initial presentation should persist for at least 4 weeks on follow-up imaging before it can be considered as PsP or TP, tumor size was measured again at the follow-up scan.

### 2.7. Response Assessment and Distinction of TP and PsP Using Histological/Immunohistochemical Analysis

Histopathological and immunohistochemical analyses were performed on the re-resected tumor specimens, when available, to establish a final diagnosis of TP or PsP in the present study. For TP, standard morphological criteria were used including increased mitotic activity, endothelial cell proliferation, and pseudopalisading necrosis. Moreover, the presence of nuclear overexpression of p53 protein in the tumor specimens confirmed TP; however, the absence of positive reactivity for p53 staining did not reject the result. On the other hand, tumor specimens with predominant treatment effects or PsP were characterized by geographic necrosis, gliosis, fibrosis, vascular hyalinization, macrophage infiltration, and dystrophic calcification using standard procedures [27]. Additionally, the Ki-67 proliferative index was calculated by determining the percentage of neoplastic cells that expressed the Ki-67 protein, avoiding regions of inflammatory cells from the tumor specimens.

### 2.8. Data Analysis

**Figure 1:**
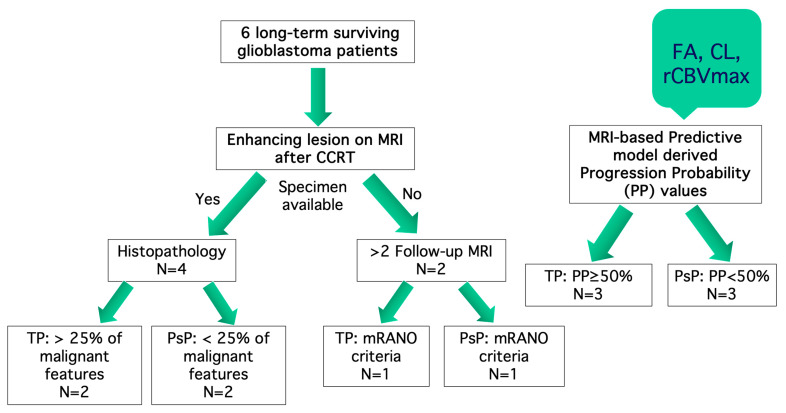
Flowchart of included patients.

These patients were dichotomized into two groups: PsP (n = 3) and TP (n = 3). When intraoperative tumor specimens were available, TP or PsP were identified by the presence of malignant features on histopathology (TP: >25% malignant features; n = 2) and PsP (<25% malignant features; n = 2) [7,28]. When tissue specimens were not available, ≥2 consecutive follow-up anatomical imaging was used to characterize PsP (n = 1) or TP (n = 1) lesions using modified RANO criteria [26], Figure 1.

In our initial work [16], the imaging parameters detailed in Section 2.5 were used in a multivariate logistic regression (LRM) analysis with backward stepwise selection, which indicated that the best classification of TP or PsP was achieved by including 3 parameters (FA, CL, and rCBV_max_). The cutoff value for the LRM was 0.55 with a sensitivity = 76%; specificity = 95%; and AUC = 0.905, based on the histological analyses. Leave-one-out cross-validation analysis revealed that 78% of cases were correctly classified by using the LRM. Therefore, we used a combination of FA, CL, and rCBV_max_, to compute the predictive probabilities (PPs) of tumor progression using the following equation:

$$f(FA,CL,\;rCBVmax)=1÷1+exp[-\left(\beta 0+\beta 1FA+\beta 2CL+\beta 3rCBVmax\right)],$$

where *β*0 = −16.17; *β*1 = 194.01; *β*2 = −285.65; and *β*3 = 1.21.

Subsequently, the PP values were used to describe each contrast-enhancing lesion as PsP or TP. The lesions were defined as PsP if the PP was <50% and TP if the PP was ≥50%.

The OS was considered the primary clinical endpoint. The survival time of all the patients was documented from the date of initial surgery to the date of death. Alive patients were censored at the time of data analysis. Each patient’s demographic and clinical information and genomic status including MGMT, *IDH* status, and OS were recorded (Table 1).

***Patient 1***

**Figure 2:**
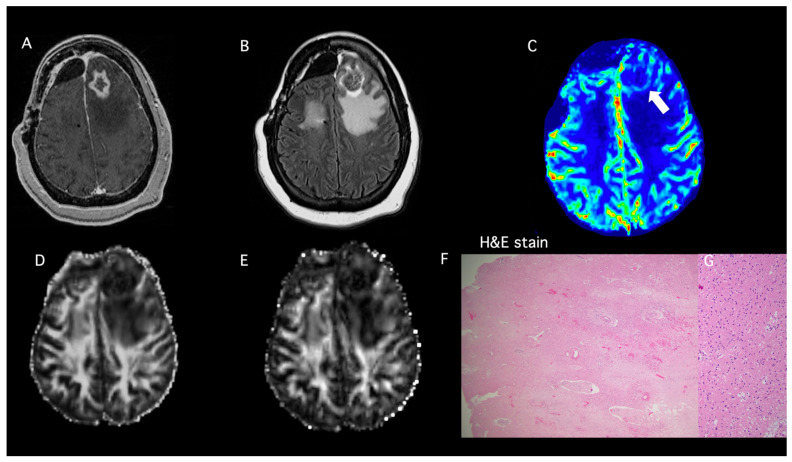
**(Patient #1):** A 50-year-old female with glioblastoma, who underwent near total resection, and received SOC therapy and was subsequently treated with mibefradil dihydrochloride combined with temozolomide. (**A**) Post-contrast T1-weighted image shows a heterogeneously enhancing lesion in the left frontal lobe. (**B**) T2-FLAIR image demonstrates hyperintense signal abnormality surrounding the lesion and extending to the posterior left frontal lobe. (**C**) DSC shows mildly elevated rCBV corresponding to the enhancing margins (white arrow). Constellation of these conventional and advanced imaging findings favored predominantly treatment-related changes (radiation necrosis). The multiparametric MRI-based predictive model comprising rCBV_max_ along with FA (**D**) and CL (**E**) suggests a diagnosis of pseudoprogression (rCBV_max_ = 1.6, FA = 0.09, CL = 0.03), with a significant component of treatment-related changes (PP = 1%). (**F**) The surgical specimen demonstrated largely geographic necrosis and radiation-induced vasculopathy consistent with treatment-related changes and (**G**) only rare infiltrative residual viable tumor cells (5% viable tumor with 95% treatment-related changes). H&E stain: hematoxylin and eosin stain.

A 50-year-old female presented with headaches and mild confusion. MRI of the brain demonstrated a heterogeneously enhancing mass with solid and cystic components in the right frontal lobe with a surrounding T2-FLAIR signal abnormality extending through the genu of the corpus callosum into the left frontal periventricular white matter. The patient underwent a near-complete resection of the tumor ten days after the first brain MRI. Histopathology confirmed the diagnosis of a glioblastoma, *IDH* wild-type, and methylated MGMT status. The patient received SOC-CCRT and subsequent treatment for one year (12 cycles) with mibefradil dihydrochloride (calcium channel antagonist) combined with temozolomide (ABTC 1101; NCT01480050). A follow-up MRI three years after the initial diagnosis demonstrated an enlarging enhancing heterogeneous mass in the left frontal lobe, associated with a confluent surrounding FLAIR signal abnormality, resulting in 5 mm rightward midline shift. Even though the mass had only a mild elevation of perfusion and the permeability metrics suggested predominantly treatment-related changes (radiation necrosis), based on the significantly increased mass effect and midline shift the lesion was resected. The surgical specimen demonstrated largely necrotic tissue consistent with treatment-related changes and minimal recurrent/residual viable neoplasm (5% residual/recurrent tumor with 95% treatment-related changes), as shown in Figure 2. Immunohistochemical analysis demonstrated negative EGFR and p53. GFAP staining showed diffuse nonspecific staining. Testing for EGFRvIII showed only wildtype EGFR reads. Ki-67 labeled some lymphocytes and occasional larger atypical nuclei consistent with a tumor. Our logistic regression model demonstrated a very low PP value of 1% (FA = 0.09, CL = 0.03, rCBV_max_ = 1.6), consistent with pseudoprogression and concordant with the histopathology. This patient is still alive, with an OS of 12.3 years; the lowest Karnofsky performance status (KPS) score recorded was 90.

***Patient 2***

A 56-year-old female presented with severe headaches. MRI of the brain demonstrated a heterogeneously enhancing mass in the right temporal lobe with surrounding T2-FLAIR signal abnormality extending into the right posterior frontal and parietal lobes. The patient underwent near-complete tumor resection two days after the first brain MRI. Histopathology revealed the diagnosis of a glioblastoma, *IDH* wild-type, with a methylated MGMT promoter. The patient received SOC-CCRT. A follow-up MRI one year and 10 months later suggested tumor progression, with a solid enhancing mass along the medial aspect of the resection cavity with surrounding FLAIR signal abnormality, and marked elevation of perfusion and permeability metrics. A surgical specimen from the repeat resection demonstrated a predominantly viable tumor with focal necrosis as well as a small foci of treatment-related changes (80% viable tumor, 15% necrosis, 5% reactive brain tissue). Our logistic regression model demonstrated a very high PP value of 99% (FA = 0.15, CL = 0.05, rCBV_max_ = 7.94), consistent with true progression and concordant with histopathology. Two years later the patient had a second tumor recurrence and received 6 cycles of bevacizumab in conjunction with temozolomide. The KPS of this patient ranged between 70 and 100. She succumbed to the glioblastoma with an OS of 5.1 years.

***Patient 3***

A 34-year-old female presented with seizures. MRI of the brain demonstrated a heterogeneously enhancing mass in the left frontal lobe with surrounding T2-FLAIR signal abnormality. The patient underwent a near-complete resection of the tumor five days after the first brain MRI. Histopathology and immunohistochemical analysis revealed the diagnosis of a glioblastoma, *IDH* wild-type, and a methylated MGMT promoter. Tumor cells were positive for GFAP and p53, with a “high level of labeling” for Ki-67, and EGFRvIII was negative. The patient received SOC-CCRT. A follow-up MRI two years later showed a large heterogeneously enhancing left frontal lobe mass with moderate elevation of perfusion and an increasing size and permeability metrics favoring a combination of viable neoplasm and treatment related changes. Pathological specimens from repeat surgery demonstrated extensive treatment-related changes and rare residual infiltrating glial tumor cells (95% treatment related changes; 5% tumor cells).

p53 strongly stained the scattered infiltrating tumor cell nuclei and Ki-67 proliferation index was up to 3% focally. Our logistic regression model demonstrated a low PP value of 1% (FA = 0.08, CL = 0.03, rCBV_max_ = 2.02), consistent with pseudoprogression and concordant with the histopathology. The patient has been treated with a combination of viral (Newcastle disease) oncolytic and dendritic cell trail vaccines since recurrence and is still alive, with an OS of 11.1 years. The KPS ranged from 70–90.

***Patient 4***

**Figure 3:**
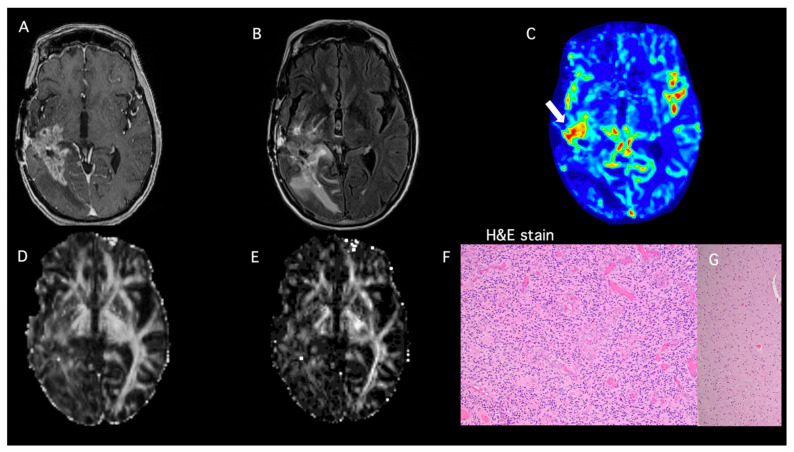
**(Patient #4):** A 57-year-old female patient with glioblastoma, who underwent near total resection, and was treated with SOC therapy. (**A**) Post-contrast T1-weighted image shows a heterogeneously enhancing mass at the margins of the resection cavity. (**B**) T2-FLAIR image demonstrates hyperintense signal abnormality surrounding the surgical margins extending to the right occipital lobe and thalamocapsular region. (**C**) DSC shows elevated rCBV from the enhancing region of the tumor (white arrow). Constellation of these conventional and advanced imaging findings favors tumor progression. The multiparametric MRI-based predictive model comprising rCBV_max_ along with FA (**D**) and CL (**E**) (rCBV_max_ = 2.02, FA = 0.21, CL= 0.09) suggests a significant component of recurrent tumor (PP = 90%). (**F**) The surgical specimen demonstrated predominantly viable tumor with (**G**) minimum necrosis and focal treatment-related changes (85% viable tumor, 2% necrosis, 13% reactive changes). H&E stain: hematoxylin and eosin stain.

A 57-year-old female presented with severe headaches. MRI of the brain demonstrated a heterogeneously enhancing mass in the right temporal lobe with surrounding T2-FLAIR signal abnormality. The patient underwent near-complete resection of the tumor six days after the first brain MRI. Histopathology revealed the diagnosis of a glioblastoma, *IDH* wild-type, methylated MGMT status, and EGFRvIII amplification. The patient received SOC-CCRT. A follow-up MRI two years later showed a new enhancing mass along the posterior margin of the resection cavity with elevated perfusion and permeability metrics indicating recurrent neoplasm. The surgical specimens from repeat surgery demonstrated predominantly viable tumors with minimum necrosis as well as focal treatment-related changes (85% tumor, 2% necrosis, 13% reactive changes), as shown in Figure 3. Immunohistochemical analysis demonstrated a Ki-67 proliferation index of up to 30%, p53 weakly to moderately labeled the majority of tumor nuclei, and EGFR showed very strong membrane expression. GFAP staining highlighted a subset of neoplastic astrocytes. Our logistic regression model demonstrated a PP value of 70% (FA = 0.21, CL = 0.09, rCBV_max_ = 2.02), consistent with true progression and concordant with the histopathology. After recurrence, the patient received additional treatment for two years with a peptide vaccine, Rindopepimut, targeting the tumor-specific EGF driver mutation, EGFRvIII (NCT01498328). The KPS of this patient ranged between 90 and 100. She had an OS of 5.2 years.

***Patient 5***

A 67-year-old female presented with headaches and lethargy with an episode of an inability to move her mouth. MRI of the brain demonstrated a heterogeneously enhancing mass in the right temporal lobe with surrounding T2-FLAIR signal abnormality. The patient underwent complete resection of the tumor two days after the first MRI. Histopathology and immunohistochemical analysis revealed the diagnosis of a glioblastoma, *IDH* wild-type, with an unmethylated MGMT promoter. The tumor cells were positive for GFAP and S100 and showed retained staining for ATRX. No fusion transcripts involving BRAF or EGFR, or aberrant transcripts of EGFR (EGFRvII or EGFRvIII), were identified. The patient received SOC-CCRT; however, adjuvant temozolomide was halted due to severe adverse events (hepatic toxicity and fever). A follow-up MRI three years later indicated tumor progression with a continued increase in tumor size over 2 months consistent with TP according to modified RANO criteria. The logistic regression model demonstrated a PP value of 99% (FA = 0.35, CL = 0.14, rCBV_max_ = 2.0), consistent with TP. Subsequently, the patient was enrolled in a clinical trial with tumor-treating fields (TTFields) and is still alive, with an OS of 6.8 years; the KPS remained at 100.

***Patient 6***

A 63-year-old female presented with palate numbness and tongue twitching. MRI of the brain demonstrated a heterogeneously enhancing mass with areas of necrosis within the posterior left frontal lobe and with involvement of the left precentral gyrus, associated with a mild surrounding T2-FLAIR signal abnormality. The patient underwent near-complete resection of the tumor five days after the first brain MRI. Histopathology and immunohistochemical analysis revealed the diagnosis of a glioblastoma, *IDH* wild-type, and a methylated MGMT promoter. A GFAP stain highlighted neoplastic astrocytes. EGFR and EGFRvIII were negative. P53 strongly stained the tumor nuclei and the Ki-67 proliferation index was up to 60%. The patient received SOC-CCRT. Follow-up MRI scans after one year demonstrated a new focus of enhancement along the posterior margin of the surgical cavity, which subsequently decreased in size over 4 months, consistent with PsP according to modified RANO criteria. The logistic regression model demonstrated a PP value of 0.1% (FA = 0.07, CL = 0.04, rCBV_max_ = 3.08), consistent with PsP. Subsequently, the patient received additional treatment for one year (12 cycles) with DCVax-L. The KPS of this patient ranged between 90 and 100. She had an OS of 5.2 years.

The summary of the multiparametric MRI-based prediction model results for each patient is found in Table 2.

## 3. Discussion

In the current study, a previously established multiparametric MRI-based prediction model [16] was used to characterize each patient’s contrast-enhancing lesions as TP or PsP in a series of six glioblastoma patients who had long-term survival outcomes. A significant concordant rate of 100% was detected between histopathology/modified RANO criteria and multiparametric MRI-derived PP values in determining the diagnosis of PsP and TP. This is a significant finding given the fact that glioblastomas are extremely heterogeneous, both phenotypically and genotypically [29,30]. An accurate assessment of treatment response can aid in optimal clinical decision making and the early introduction of therapy, which could, in turn, improve survival outcomes [12,23].

Glioblastoma is a deadly primary brain neoplasm, with rare long-term survivors [13,31]. The average life expectancy of glioblastoma patients after diagnosis is 15 to 17 months, and only <5% of these patients survive for at least 5 years [13], and are considered as long-term survivors [32,33]. Additionally, extreme long-term-surviving patients, living for 10 years or more after diagnosis, comprise only less than 1% of all patients [34]. To improve survival/clinical outcomes, an accurate diagnosis of post therapeutic TP is crucial because it impacts clinical decision, allowing for early interventions and determining overall prognosis. Patients with TP often require repeat biopsy/surgical resection and/or switching to alternative therapies such as tumor-treating fields (TTFields), chemowafers, antiangiogenic therapy, or immunotherapy. On the other hand, patients with PsP are symptomatically managed with a continuation of adjuvant temozolamide and regularly tracked with short-term follow-up MRI scans [35,36,37].

Conventional neuroimaging often fails to distinguish between TP and PsP, as both conditions can demonstrate new or increasing enhancement within the radiation field and a progressive enlargement of T2/FLAIR signal abnormalities [38,39]. These changes reflect blood–brain barrier impairment and are nonspecific, being seen in both TP and PsP. Previous studies used DTI and DSC-PWI independently to differentiate TP from PsP, with variable accuracies (62% to 91%) [15,16,40,41,42,43]. Intratumoral heterogeneity in glioblastomas causes mismatched findings across different neuroimaging parameters due to variations in tumor biology, such as cellularity, metabolism, angiogenesis, or immunogenicity. This variability affects MRI features and influences tumor growth and treatment response, making a single imaging technique insufficient for reliable evaluation [12]. Our study reinforces the notion that multiparametric analysis, which combines the strengths of various neuroimaging techniques, provides a more accurate assessment of treatment response in glioblastoma patients [14]. Specifically, integrating DTI (FA, CL) and DSC-PWI (rCBV_max_) parameters offers a more reliable evaluation of tumor biology and the microenvironment. We observed a 100% concordance between multiparametric MRI-derived PP values and histopathology/modified RANO in three TP and three PsP cases. In an era of personalized medicine, these findings suggest that a multiparametric MRI approach could be useful not only in early patient stratification (TP or PsP) but also in dynamic treatment monitoring.

In the current study, we also sought to uncover molecular and clinical indicators that could identify long-term survivors of glioblastoma. In agreement with the current definition of a glioblastoma (2021 WHO classification of CNS tumors), we only incorporated *IDH* wild-type tumors [44], while previous studies with larger cohorts have used outdated definitions with patients who would now be diagnosed with tumors with better survival, such as an *IDH* mutated tumor or even an oligodendroglioma [13,45]. The status of MGMT promoter methylation is well-established as a positive prognostic factor in glioblastomas [46,47]. Indeed, five of the six patients with a glioblastoma in our study harbored MGMT promoter methylation (patients #1, #2, #3, #4 and #6), further emphasizing the clinical importance of determining MGMT promoter status as a prognostic indicator in neurooncology. In addition, all PsP patients (patients #1, #3 and #6) were also MGMT promoter methylated, reinforcing their association with, as well as their responsiveness to, temozolomide [48].

Other factors that affect the overall survival of patients with glioblastoma include age at diagnosis, gender, functional status, extent of surgical resection, and association with alternative targeted therapies. The median age at diagnosis was 56 years in our patient population, a younger median age at diagnosis than the 65 years reported for the general glioblastoma population. Younger patients have a better outcome [49], due to fewer comorbidities, but there are also more favorable molecular and genetic alterations in younger patients [50]. A prior study showed that for every 4.7 years younger the age at diagnosis was, the OS was one year longer after 10 years of survival [34]. Interestingly our extreme survivors (patient #1, 12.3 years and patient #3, 11.1 years) were the youngest patients in our cohort, with ages 50 and 34 years at the time of initial diagnosis, respectively, and are still alive. The KPS recorded in our glioblastoma patients varied from 70 to 100; thereby, we included patients with a high functioning status, a known prognostic variable. In terms of the extent of surgical resection, all patients had at least near-total resection (95–99.9% contrast enhancing tumor reduction ∣+∣ ≤ 1 cm^3^ residual contrast enhancing tumor) [51]; for instance, 12-month progression-free survival (PFS) is increased by 50% after complete tumor resection [49].

While epidemiological data have indicated that the incidence of glioblastoma is 1.6 times higher in males compared to females [52], all of the patients in our series were female. A prior study using radiographical and transcriptomics data suggested that female patients with a glioblastoma have improved survival due to distinct molecular mechanisms [53]. In addition, several other studies indicated that estrogen could protect against the development of glioblastoma and promote favorable pathophysiology [54,55,56,57]. Seizures have been described to lead to earlier presentation and imaging analysis, representing a leading bias for long-term survivors of glioblastomas [58,59,60]. However, only one patient in our cohort had seizures at presentation and all patients had surgery just a few days after the initial brain MRI. It is logical that early-stage disease diagnosis and prompt tumor resection improve survival rather than the type of initial symptom. In addition, seizures are a common presentation of low-grade gliomas [61].

The current SOC for treatment of glioblastoma since 2005 is based on the landmark trial of radiotherapy plus concomitant and adjuvant temozolomide [62]. Indeed, in our cohort, all patients were treated with concomitant CCRT, and only one patient was not able to complete the full course of adjuvant temozolomide. Nevertheless, a recent meta-analysis showed no apparent improvement in 5-year survival post-initiation of multimodal therapy [13]. This observation may indicate a molecularly distinct group of patients within this heterogeneous disease. Most recently, alternative treatment modalities, such as TTFields, chemowafers, anti-angiogenic therapy, or clinical trials, including immunotherapy, are under active investigation as an attempt to improve survival in patients with glioblastoma. Notably, all patients in our cohort received experimental therapy and/or additional FDA-approved therapy (TTFields). One patient received a calcium channel antagonist agent (patient #1); three patients received immunotherapy, including a combination of a viral (Newcastle disease) oncolytic and dendritic cell vaccine (patient #3), a peptide vaccine, Rindopepimut (patient #4), and a dendritic cell vaccine (patient # 6); one received TTFields (patient #5); and one received bevacizumab, an antiangiogenic therapy (patient # 2). Our results are consistent with prior findings and with clinical trials and substantiate the concept that patients with glioblastomas treated with immunotherapy [63,64] and TTFields [65,66,67,68] generally exhibit longer-term tails in survival curves.

There are limitations related to the multiparametric MRI-based prediction model. The ability to discriminate accurately was 75.7% in differentiating PsP from TP in our validation study [17]. The prediction model could potentially be improved by combining the analysis of DTI and DSC-PWI data from both the contrast enhancing and the peritumoral regions along with incorporation of molecular data (e.g., MGMT methylation, *IDH* mutational status) and clinical indicators into a multivariate regression analysis. Moreover, a larger patient cohort with comprehensive clinical and molecular data could facilitate the identification of novel genetic factors to distinguish this rare subset of patients with exceptional survival. These insights could enhance our understanding of tumor biology and uncover therapeutic vulnerabilities, potentially leading to improved treatments for the broader glioblastoma patient population.

Due to a small sample size, we did not perform statistical analyses to establish any relationships among demographic and molecular biomarkers and survival outcomes in the current study.

### Future Directions

Artificial intelligence (AI) is a rapidly advancing field with significant potential to enhance the precision of both diagnostic and therapeutic approaches. Radiomic, radiogenomic, and radiopathomic techniques offer noninvasive means of assessing the tumor microenvironments, enabling dynamic and detailed evaluations of the regional heterogeneity of brain tumors [28,69,70]. Studies have shown that machine learning based models can effectively differentiate TP from PsP. In particular, the multiparametric radiomics model outperforms single-parametric models, incorporating data from structural MRI, DTI, and DSC-PWI parameters [10,71]. These results highlight the necessity of multiparametric approaches, even when utilizing advanced machine learning techniques, to achieve superior outcomes compared to traditional logistic regression models [14].

## 4. Conclusions

Our multiparametric MRI-based prediction model has the capability to identify an inherently prognostic tumor characteristic by accurately distinguishing TP from PsP. This distinction significantly impacts patient management and clinical outcomes for months and even years post-diagnosis by enabling early and appropriate therapeutic interventions. However, our findings require further validation in future studies including larger patient populations.

## Figures and Tables

**Table 1:** Demographic and molecular characteristics of long-term glioblastoma survivors.

Patient ID	Gender	Age at Initial Diagnosis (Years)	KPS Score	Surgery	MGMT	SOC Treatment Completed	Additional Treatment	OS (Years)
1	F	50	90	Near total resection	+	yes	Calcium channel antagonist	12.3
2	F	56	70–100	Near total resection	+	Yes	Antiangiogenic therapy	5.1
3	F	34	70–90	Near total resection	+	Yes	Immunotherapy	11.1
4	F	57	90–100	Near total resection	+	Yes	Immunotherapy	5.2
5	F	67	100	Complete resection	-	Yes	Tumor-Treating Fields	6.8
6	F	63	90–100	Near total resection	+	Yes	Immunotherapy	5.2

Abbreviations: KPS: Karnofsky performance status; MGMT: O^6^-methylguanine-DNA methyltransferase; SOC: standard of care; OS: overall survival. Please note, + indicates MGMT promoter methylation and - indicates MGMT promoter unmethylation status.

**Table 2:** Summary of results.

Patient ID	DTI-FA	DTI-CL	DSC-rCBV_max_	PP-Value TP ≥ 50% PsP < 50%	Histopathology	Modified RANO
1	0.09	0.03	1.6	1%	PsP	
2	0.15	0.05	7.94	99%	TP	
3	0.08	0.03	2.02	1%	PsP	
4	0.21	0.09	2.02	70%	TP	
5	0.35	0.14	2.0	99%		TP
6	0.07	0.04	3.08	0.1%		PsP

Abbreviations: FA: fractional anisotropy; CL: coefficient of linear anisotropy; PP: predictive probability of tumor progression, rCBV_max_: maximum value of relative cerebral blood volume; PsP: pseudoprogression; TP: true progression; RANO: response assessment in neuro-oncology.

## Data Availability

The data that support the findings of this study are available from the corresponding author, [S.C.], upon reasonable request. The data are not publicly available due to privacy and ethical restrictions.

## Abbreviations

CAR-T—chimeric antigen receptor T cell therapy; CCRT—concurrent chemoradiation therapy; CL—coefficient of linear anisotropy; CP—coefficient of planar anisotropy; CS—coefficient of spherical anisotropy; DSC—dynamic susceptibility contrast; FA—fractional anisotropy; *IDH*—isocitrate dehydrogenase; MD—mean diffusivity; MGMT—methylguanine–DNA–methyltransferase; MPRAGE—magnetization-prepared rapid acquisition of gradient echo; PP—predictive probability of tumor progression; PsP—pseudoprogression; PWI—perfusion-weighted imaging; rCBV_max_—maximal relative cerebral blood volume; SOC—standard of care; TP—true progression; TTFields—tumor-treating fields.
